# The Crosstalk Between YAP/TAZ and Cancer Metabolism: From Mechanistic Insights to Drug Discovery

**DOI:** 10.3390/ijms27177537

**Published:** 2026-08-23

**Authors:** Yinhuang Gao, Minghong Chen, Songxia Lin, Min Huang, Jing Jin

**Affiliations:** 1 School of Pharmaceutical Sciences, Sun Yat-sen University, Guangzhou 510006, China; 13413986992@163.com (Y.G.); 13380622553@163.com (M.C.); linsongxia2023@163.com (S.L.); huangmin@mail.sysu.edu.cn (M.H.); 2 Institute of Clinical Pharmacology, Sun Yat-sen University, Guangzhou 510006, China; 3 Guangdong Provincial Key Laboratory of New Drug Design and Evaluation, School of Pharmaceutical Sciences, Sun Yat-sen University, Guangzhou 510006, China

**Keywords:** YAP/TAZ, cancer metabolism, metabolic reprogramming, therapeutic target

## Abstract

As the core transcriptional co-activators of the Hippo signaling pathway, YAP and TAZ play essential roles in maintaining tissue homeostasis and in tumorigenesis. Their aberrant activation is frequently observed in human malignancies, and accumulating evidence has identified them as crucial drivers of tumor initiation and progression. YAP/TAZ have been recently recognized as key regulators of cellular metabolic reprogramming, a hallmark of cancer that fuels tumor cell proliferation by rewiring glucose, lipid, amino acid, and nucleotide metabolism. Conversely, the activity of YAP/TAZ is modulated by metabolites such as glucose and lipids, establishing a complex bidirectional regulatory circuit. Therefore, deciphering this intricate crosstalk is of great importance for cancer therapy and drug discovery. In this review, we systematically clarify the interplay between YAP/TAZ and metabolic reprogramming in cancer, delineate the core molecular networks through which YAP/TAZ govern each metabolic pathway, and summarize the current pharmacological inhibitors targeting YAP/TAZ-regulated metabolic networks. Collectively, these findings pave the way for therapeutic approaches targeting YAP/TAZ-driven metabolic vulnerabilities in cancer.

## 1. Introduction

Cancer is one of the leading causes of morbidity and mortality worldwide [1]. Cancer metabolic reprogramming represents a core hallmark of malignancy. By rewiring glycolysis, de novo fatty acid synthesis, glutaminolysis, and nucleotide metabolism, it provides tumor cells with the energy, biosynthetic building blocks, and the ability to maintain redox balance required for rapid proliferation [2,3]. This metabolic remodeling is not merely a survival strategy for adaptation to hypoxic and nutrient-depleted microenvironments; it also creates unique “metabolic vulnerabilities” that have emerged as key therapeutic targets [4,5]. At the molecular level, several key regulators orchestrate the metabolic reprogramming in cancer cells. Thus, understanding the complex relationship between cancer metabolic reprogramming and its regulators is essential for anti-cancer drug development.

Among the oncogenic pathways, the Hippo signaling pathway plays a particularly prominent role in tumorigenesis. Yes-associated protein (YAP) and transcriptional coactivator with PDZ-binding motif (TAZ) are the core transcriptional co-activators of the Hippo signaling. Activation of YAP/TAZ is a central event regulating cell proliferation, differentiation and stemness. Abnormal activation of YAP/TAZ frequently occurs in various cancers such as lung cancer, hepatocellular carcinoma, and colorectal cancer [6,7,8], and is always associated with poor prognosis [9,10].

In recent years, emerging evidence underscores the pivotal role of YAP/TAZ in the regulation of cancer metabolic reprogramming [3]. Beyond this, YAP and TAZ are also regulated by metabolites [11]. Although substantial progress has been made in deciphering the molecular mechanisms underlying the YAP/TAZ–metabolism interaction, most findings remain at the preclinical stage [12,13]. Recent advances in the development of TEAD inhibitors, particularly compounds targeting TEAD auto-palmitoylation or disrupting the YAP/TAZ–TEAD transcriptional complex, have demonstrated promising anti-tumor activity in early clinical studies [14]. However, challenges including patient selection, biomarker identification, tumor heterogeneity, and acquired resistance continue to limit the clinical translation of YAP/TAZ-targeted therapies [15]. Therefore, a comprehensive understanding of the dynamic interaction between YAP/TAZ signaling and metabolic remodeling is essential for developing precision therapeutic strategies.

However, several critical challenges remain, including the mechanistic dissection of how upstream metabolic signals differentially modulate YAP/TAZ activity, the optimization of combination therapeutic strategies targeting the YAP/TAZ-metabolism axis, and the translation of preclinical findings into precise clinical regimens. This review systematically delineates the core molecular networks through which YAP/TAZ govern each metabolic pathway, summarizes the feedback regulation of YAP/TAZ by metabolic factors, and evaluates current pharmacological inhibitors and combination strategies in preclinical and clinical development, aiming to provide a comprehensive framework for future research and drug discovery.

## 2. YAP/TAZ Regulate Glucose Metabolism in Cancer

Glucose serves as the primary energy source and biosynthetic building block essential for the survival and growth of tumor cells. In highly proliferative cancer cells, aberrant glucose uptake and enhanced glycolysis represent core metabolic hallmarks that supply energy and metabolic intermediates necessary for rapid proliferation [11,16]. YAP/TAZ, as key transcriptional coactivators, play a crucial regulatory role in this metabolic process [17].

YAP/TAZ ensure an adequate glucose supply for tumor cells by regulating the expression and function of glucose transporters (GLUTs). GLUT1, the most representative member of the GLUT family, is highly expressed in various tumor cells, with its expression level positively correlated with both the glucose uptake capacity of these cells and the intensity of the Warburg effect. This correlation makes GLUT1 a central target through which YAP/TAZ regulate glucose uptake. In endometrial cancer cells, YAP/TAZ bind to TEAD transcription factors to directly activate GLUT1 promoter activity. Notably, YAP/TAZ directly activate GLUT1 promoter activity via TEAD binding in endometrial cancer [18], and drive GLUT1 transcription in pancreatic cancer to enhance glucose uptake [19]. Beyond GLUT1, YAP/TAZ also regulate other GLUT family members. In colorectal cancer, YAP upregulates GLUT3 expression by activating the AKT signaling pathway, thereby enhancing glucose uptake [20]; in hepatocellular carcinoma, under high-glucose conditions, the YAP-carbohydrate response element-binding protein (ChREBP) complex promotes GLUT5 expression, providing tumor cells with a fructose uptake route and increasing metabolic flexibility [21]. In addition to transcriptional regulation, YAP/TAZ modulate GLUT function through post-translational modifications: YAP promotes AKT-mediated phosphorylation of GLUT1 at serine 490, facilitating its translocation to the plasma membrane, and upregulates human antigen R (HuR) to stabilize GLUT1 mRNA, thereby enhancing glucose uptake capacity [19,22].

In addition to ensuring glucose uptake, YAP/TAZ further regulate the activities of multiple key glycolytic enzymes to enhance glycolytic flux. As a rate-limiting enzyme in glycolysis, hexokinase 2 (HK2) is a core target of YAP regulation. In pancreatic and gastric cancer cells, YAP directly promotes HK2 transcription [16,23]; in colorectal cancer, YAP enhances HK2 catalytic activity via AKT2-mediated phosphorylation at Thr473, thereby promoting glycolytic flux and increasing lactate production [24]. Notably, HK2 can also act as a scaffold protein to promote glycogen synthase kinase 3 beta (GSK3β) phosphorylation and stabilize SNAIL, thus linking YAP-mediated metabolic regulation with epithelial–mesenchymal transition and enabling coordinated control of metabolic reprogramming and tumor metastasis [25]. The expression and activity of another rate-limiting enzyme, pyruvate kinase M2 (PKM2), are also precisely regulated by YAP/TAZ. During nuclear translocation, YAP cooperates with hypoxia-inducible factor 1α (HIF-1α) to bind hypoxia response elements in the PKM2 promoter, directly upregulating PKM2 transcription and promoting its tetramer formation, thereby enhancing enzymatic activity [26]. In gastric cancer cells, YAP1 stabilizes PKM2 protein through ubiquitin-specific protease 4 (USP4)-mediated deubiquitination, further increasing glycolytic output [27]. Beyond HK2 and PKM2, YAP/TAZ also regulate the expression and activity of other glycolytic enzymes, including phosphofructokinase 1 (PFK1), 6-phosphofructo-2-kinase/fructose-2,6-bisphosphatase 3 (PFKFB3), and lactate dehydrogenase A (LDHA), thereby coordinating glycolytic flux at multiple steps [16,22,23].

Notably, beyond the shared YAP/TAZ regulatory mechanisms described above, TAZ has been independently implicated in driving aerobic glycolysis through a TAZ-Notch1 positive feedback loop in lung cancer. In this context, TAZ enhances Notch1 transcriptional activity in a TEAD1-independent manner, while Notch1 reciprocally promotes glycolytic gene expression, leading to excessive lactate production that suppresses cytotoxic T cell activity and facilitates immune evasion [28].

**Figure 1:**
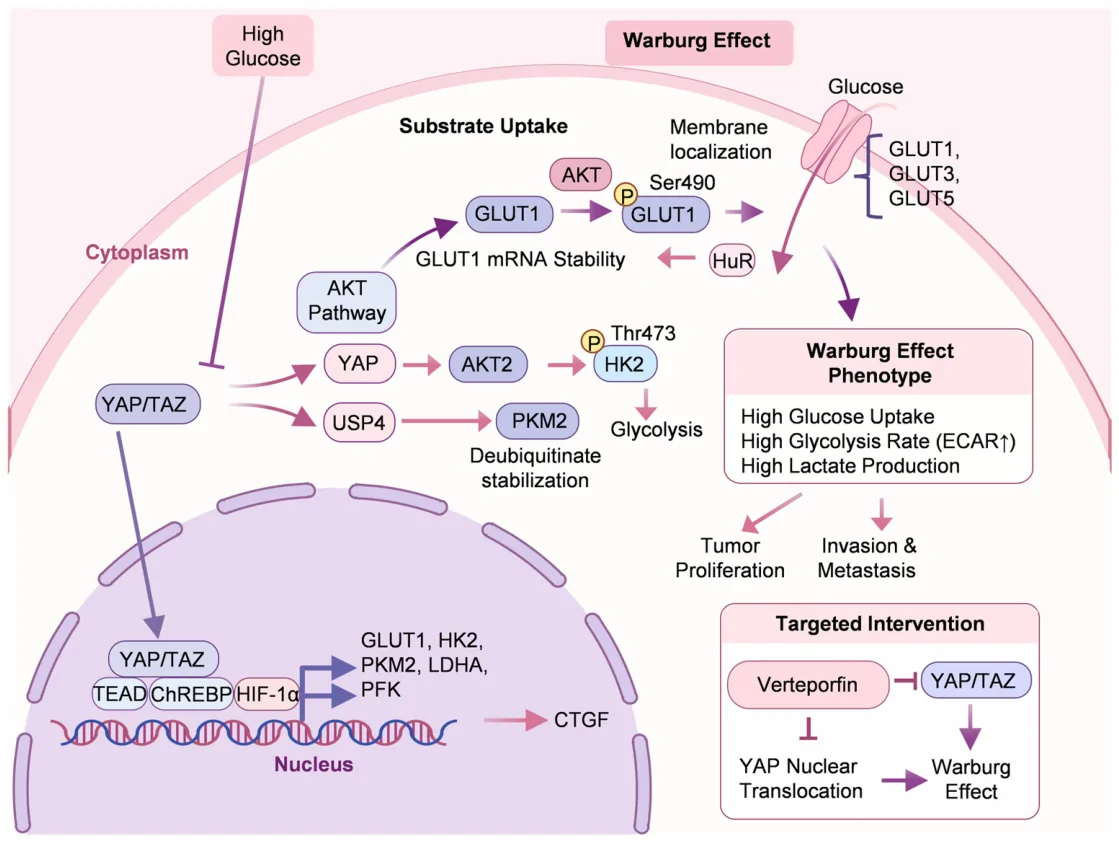
YAP/TAZ-mediated regulation of glucose metabolism in cancer. YAP/TAZ promote glucose uptake by enhancing GLUT1 membrane localization (AKT-mediated) and mRNA stability (HuR-mediated). YAP activates AKT2, which phosphorylates HK2 (Thr473) to initiate glycolysis. This cascade drives high glucose uptake, glycolysis, and lactate production, supporting tumor proliferation and invasion. Pharmacological inhibitors (verteporfin) disrupt YAP nuclear translocation and reverse the Warburg effect.

Taken together, these multi-level regulatory mechanisms establish a robust YAP/TAZ-driven program that fuels rapid tumor cell proliferation by coordinating glucose uptake and glycolytic enzyme activities across the entire process, from glucose supply to metabolic utilization (Figure 1).

## 3. YAP/TAZ Regulate Lipid Metabolism in Cancer

Tumor cells undergo reprogramming of lipid metabolism to adapt to their microenvironment, maintain malignant proliferation, and promote metastasis, with dysregulated remodeling of fatty acid and cholesterol metabolism playing a particularly pivotal role. Through the modulation of key metabolic enzyme expression, YAP/TAZ function as a central regulatory nexus in the reprogramming of tumor lipid metabolism, thereby supplying critical support for energy production, biosynthetic requirements, and adaptation to the microenvironment.

In fatty acid metabolism, YAP/TAZ regulate both core processes: de novo synthesis and β-oxidation. During de novo fatty acid synthesis, YAP acts as a coactivator of sterol regulatory element-binding proteins (SREBPs), forming complexes with SREBP-1c or SREBP-2 that directly bind to promoter regions of key genes such as fatty acid synthase (FASN) and acetyl-CoA carboxylase (ACC). This binding promotes gene transcription and enhances fatty acid synthesis capacity [29,30,31]. In breast cancer, YAP1 activation directly upregulates FASN transcription, whereas acyl-CoA synthetase long-chain family member 3 (ACSL3) suppresses YAP1 activity and nuclear translocation by inhibiting YES1 phosphorylation, thereby indirectly repressing FASN expression and lipid synthesis [30]; in colorectal cancer, YAP1 knockdown significantly reduces both the mRNA and protein levels of FASN and ACC1, thereby inhibiting de novo fatty acid synthesis [29]. YAP upregulates serum/glucocorticoid-regulated kinase 1 (SGK1) expression via TEAD transcription factors at the level of signaling crosstalk. SGK1 in turn activates the mTORC1/SREBP pathway, promoting SREBP maturation and nuclear translocation, and ultimately upregulates multiple lipogenic genes, including FASN and stearoyl-CoA desaturase (SCD) [32].

In addition to regulating fatty acid synthesis, YAP/TAZ enhance the metabolic adaptability of tumor cells under nutrient-deprived conditions by selectively activating the expression of genes involved in fatty acid oxidation (FAO). In tumor cells undergoing lymph node metastasis, YAP is selectively activated and directly upregulates the transcription of carnitine palmitoyltransferase 1A (CPT1A) and enzymes required for fatty acid β-oxidation, thereby markedly increasing FAO activity [33].

Dysregulated cholesterol metabolism is likewise a critical component of tumor metabolic reprogramming, as tumor cells enhance cholesterol synthesis or uptake to support membrane biosynthesis and signal transduction. In colorectal cancer, YAP directly drives the transcription of zinc finger MYND-type containing 8 (ZMYND8) by binding to TEAD-binding sites in the ZMYND8 promoter. ZMYND8 then cooperates with sterol regulatory element-binding protein 2 (SREBP2) to activate the expression of key cholesterol synthesis genes, such as 3-hydroxy-3-methylglutaryl-CoA reductase (HMGCR) and squalene epoxidase (SQLE), by enhancing chromatin compaction and recruiting the Mediator complex [34]. Notably, tumor cells with high YAP activity rely more heavily on endogenous cholesterol synthesis and exhibit significantly greater sensitivity to statins (e.g., atorvastatin) than YAP-low tumor cells, suggesting that the YAP-regulated cholesterol synthesis pathway may serve as a viable therapeutic target [34].

In addition to the YAP-mediated lipid metabolic reprogramming discussed above, emerging evidence suggests that TAZ also independently regulates fatty acid synthesis in tumor cells. In lung cancer, KLF5 cooperates with TAZ to directly bind the FASN promoter and enhance its transcription, leading to increased de novo fatty acid synthesis and release of free fatty acids into the tumor microenvironment. These fatty acids are subsequently taken up by regulatory T cells (Tregs) via CD36, promoting their fatty acid oxidation-dependent differentiation and contributing to an immunosuppressive milieu [35]. This KLF5-TAZ-FASN axis provides one of the few direct demonstrations of TAZ-specific regulation of tumor lipid metabolism and highlights its unique role in coupling metabolic reprogramming with immune evasion.

**Figure 2:**
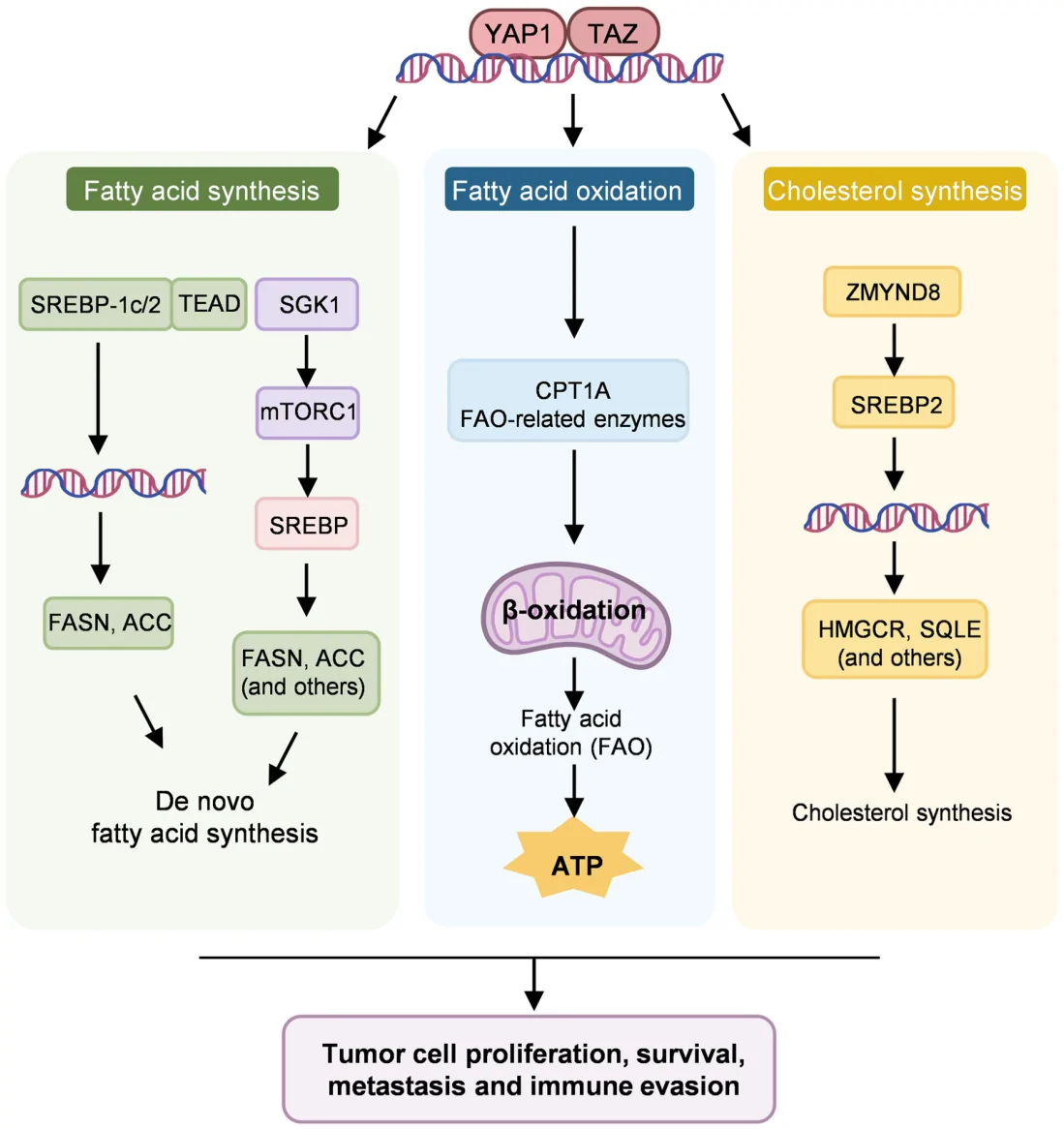
YAP/TAZ-mediated regulation of lipid metabolism in cancer. YAP/TAZ regulate tumor lipid metabolic remodeling by controlling fatty acid synthesis, fatty acid oxidation, and cholesterol biosynthesis. YAP promotes de novo fatty acid synthesis through activation of SREBP-dependent lipogenic programs and TEAD-mediated signaling pathways, leading to increased expression of key enzymes such as FASN, ACC, and SCD. YAP also enhances fatty acid oxidation by inducing CPT1A and β-oxidation-related enzymes to support energy metabolism. In cholesterol metabolism, YAP activates the ZMYND8–SREBP2 axis to promote transcription of cholesterol biosynthetic genes, including HMGCR and SQLE. In addition, TAZ cooperates with KLF5 to induce FASN expression, increasing free fatty acid production and promoting CD36-dependent fatty acid uptake and differentiation of regulatory T cells, thereby contributing to an immunosuppressive tumor microenvironment. Collectively, YAP/TAZ-driven lipid metabolic reprogramming supports tumor cell proliferation, survival, metastasis, and immune evasion.

Collectively, YAP/TAZ coordinately regulate fatty acid synthesis, fatty acid oxidation, and cholesterol metabolism through multiple targets and signaling pathways, thereby providing comprehensive metabolic support for tumor cell proliferation, survival, and metastasis (Figure 2).

## 4. YAP/TAZ Regulate Amino Acid Metabolism in Cancer

Amino acid metabolism represents a critical pathway through which tumor cells acquire nitrogen, synthesize proteins and nucleotides, and maintain redox balance. It primarily encompasses glutamine metabolism, serine–glycine one-carbon metabolism, and the metabolism of other amino acids such as proline and aspartate [36]. As core transcriptional coactivators, YAP/TAZ construct a tumor-specific amino acid metabolic network by targeting key metabolic enzymes and transporters. This network is functionally conserved across multiple tumor types, including breast cancer, liver cancer, and colorectal cancer, and is closely associated with tumor proliferation, metastasis, and microenvironmental adaptation [37,38].

As a major donor of nitrogen and carbon for tumor cells, glutamine is extensively reprogrammed in a manner central to cancer progression [11]. YAP/TAZ enhance glutamine uptake by upregulating the expression of glutamine transporters. In colorectal cancers with KRAS mutations, YAP1/TAZ markedly upregulate the glutamine transporters SLC1A5 and SLC38A2, thereby activating mTOR signaling and driving proliferation [39]. In hepatocellular carcinoma, YAP/TAZ directly control SLC38A1 transcription; knockdown of SLC38A1 significantly reduces glutamine consumption, whereas its ectopic re-expression rescues the uptake deficit caused by YAP/TAZ loss [40]. In metastatic tumor cells, YAP/TAZ modulate glutamine uptake by regulating SLC1A5 expression, a process closely intertwined with c-MYC signaling [41]. In addition to enhancing uptake, YAP/TAZ also selectively regulate glutamine-metabolizing enzymes. For instance, they promote glutamine metabolism by inducing transaminases such as GOT1 and phosphoserine aminotransferase 1 (PSAT1). Chromatin immunoprecipitation assays have confirmed that the TAZ/YAP complex binds directly to an enhancer region of GOT1, enabling precise transcriptional control [42]. In liver tissue, YAP directly upregulates the expression and activity of GLUL, thereby promoting glutamine synthesis to support nucleotide biosynthesis [37]. Similarly, in LKB1-deficient breast cancer, YAP1/TAZ enhance both GLUL expression and activity while also upregulating transporters such as SLC38A1, collectively elevating intracellular glutamine levels [43].

In addition to supplying biosynthetic precursors to tumor cells, serine and glycine metabolism helps maintain redox balance and regulate one-carbon metabolism. YAP/TAZ modulate serine production by targeting key enzymes in the serine synthesis pathway (SSP). In colorectal cancer cells tolerant to serine starvation, the SSP enzymes phosphoglycerate dehydrogenase (PHGDH), PSAT1, and phosphoserine phosphatase (PSPH) are significantly upregulated, and PHGDH expression correlates with advanced TNM stage and poor prognosis [44]. In lung cancer cells, suppression of nuclear GSK3 signaling allows YAP/TAZ to relieve inhibition of serine biosynthetic enzymes such as PHGDH and PSAT1, thereby promoting de novo serine synthesis [36]. Furthermore, the transcription factor YY2 binds the PHGDH promoter to repress its transcription, and YAP/TAZ may indirectly affect serine synthesis by modulating YY2 signaling—a regulatory axis considered important for serine metabolic reprogramming in tumor cells [45]. Glycine derived from serine metabolism can enter one-carbon metabolism via serine hydroxymethyltransferase 2 (SHMT2)-mediated reactions, providing carbon units for nucleotide synthesis. By regulating PHGDH and PSAT1 expression, YAP/TAZ may thus indirectly influence one-carbon flux and nucleotide biosynthetic capacity [36].

**Figure 3:**
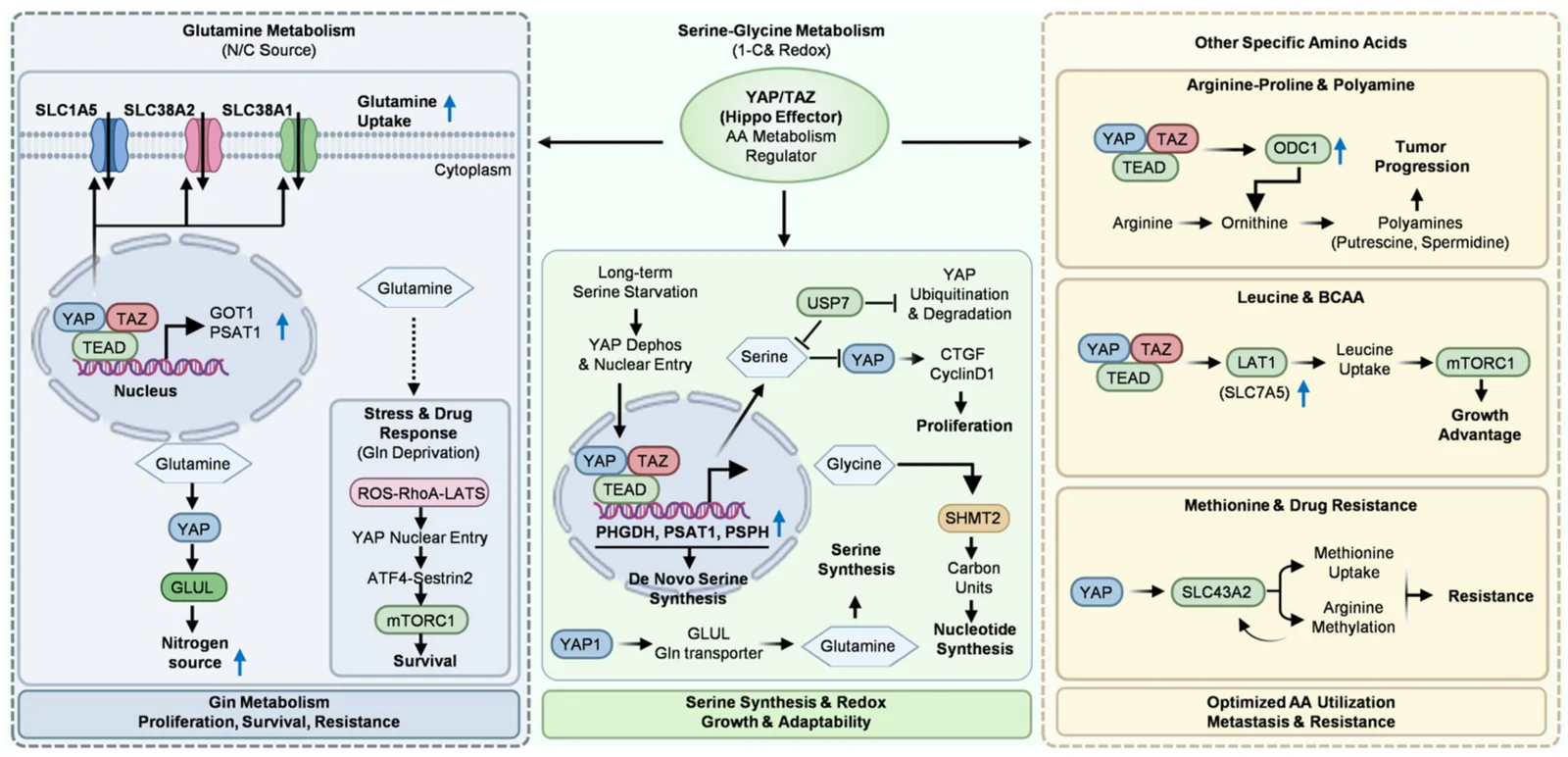
YAP/TAZ-mediated regulation of amino acid metabolism in cancer. YAP/TAZ-TEAD complexes upregulate glutamine transporters (SLC1A5, SLC38A2, SLC38A1) and drive transcription of GOT1/PSAT1/GLUL, promoting glutamine anaplerosis for TCA cycle energy and biosynthesis. Conversely, glutamine deprivation can activate YAP through a ROS-RhoA-LATS axis (see Section 7 for details). Long-term serine starvation triggers YAP nuclear translocation; YAP/TAZ-TEAD upregulate PHGDH/PSAT1/PSPH for de novo serine synthesis. Serine stabilizes YAP via USP7-mediated deubiquitination, boosting CTGF/CyclinD1 for proliferation. Glycine from serine supplies one-carbon units via SHMT2 for nucleotide synthesis. YAP/TAZ upregulate ODC1 to drive polyamine synthesis, supporting progression. YAP/TAZ induce LAT1 (SLC7A5) to enhance leucine uptake, activating mTORC1 for growth. YAP regulates SLC43A2 to boost methionine uptake, linking to drug resistance via arginine methylation.

Additionally, YAP/TAZ promote the polyamine synthesis pathway in arginine and proline metabolism. They transcriptionally activate ornithine decarboxylase 1 (ODC1), the rate-limiting enzyme of polyamine biosynthesis, thereby driving the conversion of arginine and ornithine into polyamines such as putrescine and spermine [46]. Metabolomic profiling of YAP-overexpressing liver tissues revealed significantly elevated levels of arginine and the polyamine synthesis intermediate N-acetylputrescine, confirming that YAP/TAZ-mediated regulation of arginine metabolism directly fuels polyamine synthesis [46]. For leucine metabolism, YAP/TAZ bind the SLC7A5 promoter via TEAD transcription factors to directly control expression of the SLC7A5/SLC3A2 (LAT1/CD98 heavy chain) transporter, thereby enhancing leucine uptake and modulating mTORC1 signaling. This regulatory mechanism is particularly critical for tumor cells to maintain a growth advantage under leucine-restricted conditions [47]. In KRAS-mutant colorectal cancer, YAP1 cooperatively upregulates amino acid transporters including SLC7A5, bolstering leucine uptake and activating downstream pathways that drive cancer progression [48]. Methionine metabolism is similarly regulated by YAP/TAZ and is linked to tumor metastasis and drug resistance. In head and neck squamous cell carcinoma, YAP knockdown markedly suppresses cysteine and methionine metabolism, thereby inhibiting metastasis, which underscores a central regulatory role for YAP in these amino acid metabolic networks [49]. YAP also influences methionine uptake by controlling expression of the methionine transporter solute carrier family 43 member 2 (SLC43A2). Moreover, an arginine methylation-dependent positive feedback loop involving SLC43A2 drives chemoresistance in tumor cells [38]. Collectively, by orchestrating the expression of amino acid transporters and metabolic enzymes, YAP/TAZ integrate glutamine metabolism, serine–glycine one-carbon metabolism, and the metabolic networks of polyamines, branched-chain amino acids, and methionine, thereby providing essential metabolic support for tumor cell proliferation, metastasis, and drug resistance (Figure 3).

## 5. YAP/TAZ Regulate Nucleotide Metabolism in Cancer

Rapidly proliferating tumor cells exhibit a dramatically increased demand for nucleotides to meet the supply of raw materials for DNA replication and RNA synthesis. Recent studies have demonstrated that YAP/TAZ play a pivotal role in maintaining nucleotide homeostasis by regulating the synthesis and catabolism of purines and pyrimidines, thereby supporting the sustained proliferation and malignant progression of tumor cells.

In the regulation of purine metabolism, YAP/TAZ participate in the synthesis and metabolic balance of purine nucleotides through multiple mechanisms. YAP can directly regulate the expression of glutamine synthetase (GLUL), promoting glutamine synthesis to provide sufficient nitrogen sources for purine synthesis. In transgenic zebrafish models, activated YAP1 significantly enhances the expression and activity of GLUL, improves intracellular glutamine homeostasis, promotes purine nucleotide production, thereby supporting the initiation and progression of hepatocellular carcinoma [37]. Under glucose-restricted conditions, YAP’s regulation of purine metabolism involves complex feedback loops. YAP also participates in purine nucleotide turnover by enhancing the activity of purine nucleoside phosphorylase (PNP), thereby accelerating purine degradation and generating D-ribose-5-phosphate (D5P), a metabolite that feeds into the pentose phosphate pathway [50]. Of note, D5P itself can function as a feedback regulator of YAP activity, a mechanism that is discussed in detail in Section 7 [50]. This mechanism maintains cellular metabolic homeostasis and ensures tumor cell survival and proliferation in nutrient-deprived environments [50]. Additionally, YAP/TAZ can indirectly participate in the PPP-mediated purine synthesis process by regulating GLUT1 expression, a mechanism highly conserved in both fish and mammals [51]. In solid tumors, YAP cooperates with the PI3K-mTORC signaling pathway to regulate purine metabolism through a phosphorylation modification network. Phosphoproteomic analysis reveals that in Luminal B breast cancer tissues, the phosphorylation level of YAP is abnormally elevated, and the purine metabolic pathway also shows significant dysregulation, suggesting that YAP may regulate the activity of key purine metabolic enzymes in a phosphorylation-dependent manner [52]. In trastuzumab-resistant HER2-positive breast cancer cells, the high expression of YAP/TAZ is closely associated with abnormal activation of purine metabolism, and inhibition of YAP/TAZ activity can reverse the drug-resistant phenotype of tumor cells and restore purine metabolic balance [53].

The regulation of pyrimidine metabolism by YAP/TAZ is achieved through direct modulation of the expression of key enzymes in the de novo pyrimidine synthesis pathway. In NF2-deficient pleural mesothelioma, loss of NF2 function activates the YAP signaling pathway, significantly upregulating the expression levels of CAD and DHODH, key enzymes in de novo pyrimidine synthesis [54]. This mechanism renders NF2-deficient tumor cells highly sensitive to pyrimidine synthesis inhibitors in both in vitro and in vivo experiments, indicating that YAP-mediated activation of pyrimidine metabolism is a core metabolic vulnerability in these tumors [54]. During tumor cells’ response to chemotherapy-induced stress, YAP/TAZ-mediated regulation of pyrimidine metabolism confers significant therapeutic resistance to tumor cells. YAP/TAZ maintain the homeostatic level of deoxyribonucleoside triphosphates (dNTPs) in cells by directly regulating the expression of key pyrimidine synthase enzymes (such as TYMS and TK1). This mechanism not only reduces tumor cell sensitivity to pyrimidine synthesis-targeted chemotherapeutic drugs, but also helps cells bypass the inhibitory effects of the RAS/MEK1 pathway in RAS-induced cellular senescence models, enabling evasion of senescence programs through sustained activation of pyrimidine synthesis [55]. In pancreatic cancer, the YAP/TAZ signaling is regulated by the GPR55 and β2-adrenergic receptor (β2-AR) signaling pathways. Inhibition of YAP/TAZ activity significantly reduces the activity of the de novo pyrimidine synthesis pathway in tumor tissues, accompanied by downregulation of HIF-1α and c-MYC expression, suggesting that YAP/TAZ may indirectly affect pyrimidine metabolism by modulating the hypoxia response pathway [56]. In hepatocellular carcinoma, YAP1 enhances glutamine synthetase (GLUL) expression and activity, increasing intracellular glutamine levels to provide nitrogen sources for pyrimidine synthesis, thereby promoting nucleotide biosynthesis [37].

It is worth noting that, to date, the mechanistic evidence for nucleotide metabolism regulation by the Hippo pathway primarily derives from YAP-driven mechanisms. Whether TAZ independently regulates purine or pyrimidine nucleotide synthesis in a YAP-independent manner remains an open question and represents an important direction for future investigation.

**Figure 4:**
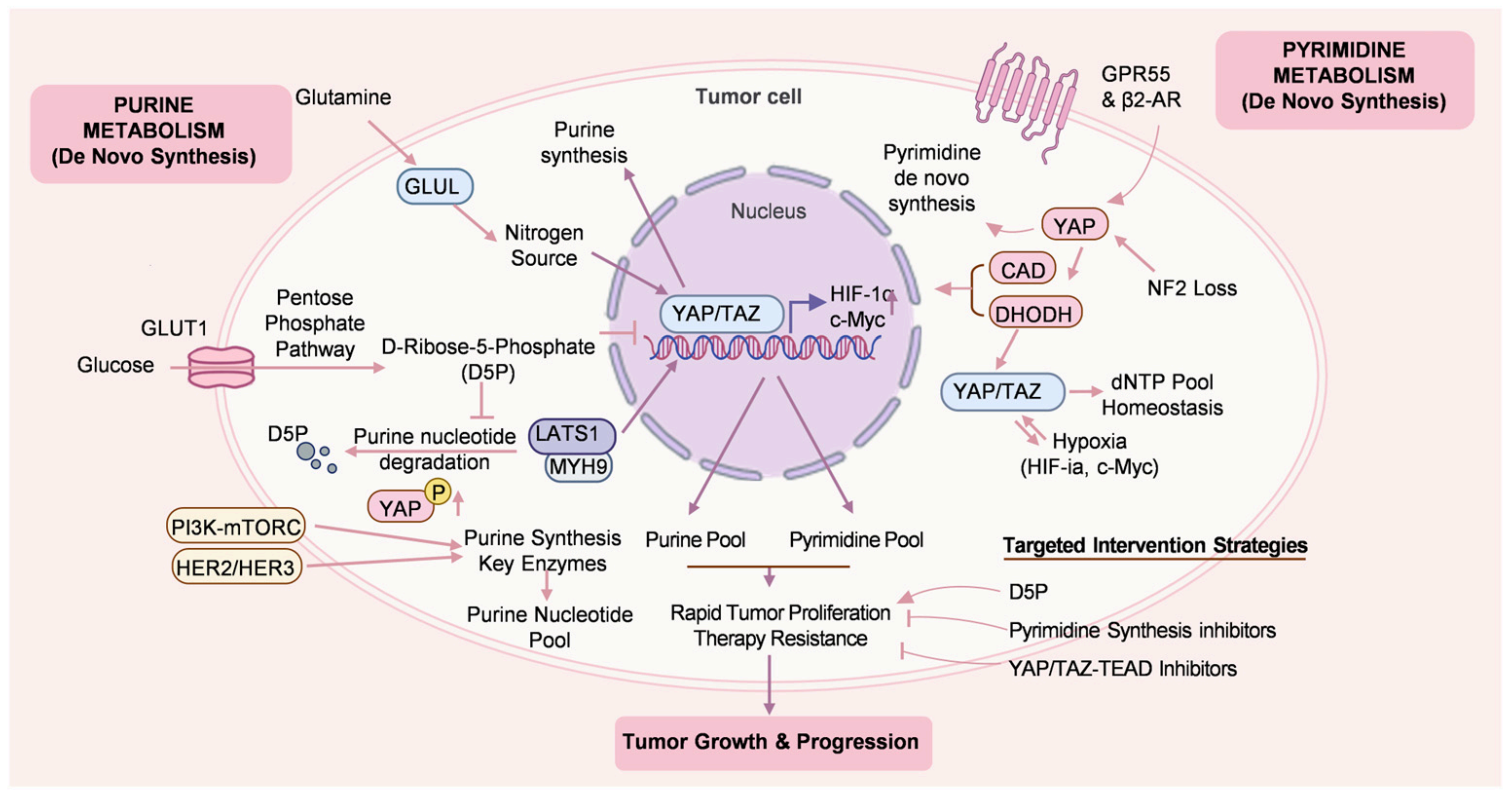
YAP/TAZ-mediated regulation of nucleotide metabolism in cancer. Purine metabolism: GLUT1-mediated glucose uptake fuels the PPP to generate D5P. GLUL utilizes glutamine to supply nitrogen for purine synthesis. YAP (regulated by PI3K-mTORC1/HER2/HER3) activates key purine synthesis enzymes, expanding the purine pool to support proliferation and therapy resistance. D5P inhibits YAP via LATS1/MYH9, forming a feedback loop that maintains metabolic homeostasis under glucose stress. Pyrimidine metabolism: GPR55/β2-AR signals modulate YAP. NF2 loss activates YAP, which upregulates de novo pyrimidine synthesis enzymes (CAD/DHODH). YAP regulates dNTP pool homeostasis via HIF-1α/c-MYC to sustain the pyrimidine pool, driving tumor growth and therapy resistance.

Under physiological conditions, YAP/TAZ participates in maintaining organ size and tissue homeostasis by regulating nucleotide metabolism. However, during tumor development, this regulatory mechanism is abnormally activated, disrupting nucleotide metabolic balance through multiple pathways to meet the demand for nucleic acid synthesis in rapidly dividing tumor cells [57]. By integrating multiple signaling pathways including PI3K-mTORC, HER2/HER3, and p53, YAP/TAZ form a multi-layered regulatory network that coordinates the dynamic equilibrium of purine and pyrimidine metabolism [52,53,54]. In Luminal B breast cancer, elevated YAP phosphorylation levels and dysregulated sphingolipid metabolism collectively influence tumor cell proliferation by modulating key nodes of purine and pyrimidine metabolic pathways, suggesting YAP/TAZ may serve as a central hub coordinating the balance of multiple metabolic processes [52]. YAP/TAZ also interact with tumor suppressors such as p53. When p53 undergoes functional mutations, its regulatory effect on YAP/TAZ-mediated nucleotide metabolism is lost, leading to abnormal accumulation of nucleotide pools in tumor cells and accelerating tumor progression [58]. Imbalanced nucleotide metabolic homeostasis is a key mechanism by which YAP/TAZ drive tumor proliferation. In chronic inflammatory microenvironments (such as asbestos-induced mesothelioma), the Hippo signaling pathway can be activated to regulate the expression of purine metabolic enzymes and promote tumor-associated inflammatory responses [59]; under glucose deprivation conditions, the YAP-mediated D5P feedback loop maintains basal purine metabolic activity and enhances tumor cell adaptation to nutrient limitations [50,51]. These findings provide a therapeutic rationale for targeting YAP/TAZ-mediated nucleotide metabolic dysregulation (Figure 4).

## 6. YAP/TAZ Regulate Mitochondrial Function in Cancer

Mitochondria, as the core of intracellular energy metabolism, play a pivotal role in the metabolic reprogramming of cancer cells. In colorectal cancer cells, abnormally overexpressed YAP inhibits Drp1 activation and its translocation to the mitochondrial membrane by suppressing JNK phosphorylation, consequently restraining excessive mitochondrial fission and reducing the release of HtrA2/Omi protein from mitochondria into the cytoplasm. This ultimately suppresses tumor cell apoptosis and promotes migration [60]. Furthermore, in NF2-deficient renal cancer cells, silencing of YAP/TAZ significantly alters mitochondrial morphology, reduces glycolysis-dependent cell growth, enhances mitochondrial respiration, and causes reactive oxygen species accumulation [61]. These findings indicate that YAP/TAZ regulate mitochondrial homeostasis by modulating mitochondrial dynamics, including the balance between fission and fusion processes, in a tumor-specific manner, thereby providing metabolic advantages for cancer cells under different microenvironmental conditions [62].

YAP/TAZ also regulate oxidative phosphorylation in the mitochondrial respiratory chain by modulating the expression of respiratory chain-related genes and the activity of metabolic enzymes, significantly impacting mitochondrial respiratory chain function and thereby reshaping cellular metabolic phenotypes. Transcriptomic analysis of clinical samples confirmed that YAP/TAZ signaling signatures are positively correlated with glycolytic gene expression and negatively correlated with oxidative phosphorylation gene expression, highlighting their pivotal role in regulating the metabolic switch of tumor cells [61]. In NF2-deficient renal tumors, YAP/TAZ silencing markedly suppresses glycolysis-dependent cell growth, enhances mitochondrial respiration, and increases reactive oxygen species levels, inducing oxidative stress-mediated cell death under nutrient stress conditions [61]. In breast cancer stem cells, ClpP agonists inhibit the function of breast cancer stem cells by suppressing the YAP signaling pathway and downregulating the activity of oxidative phosphorylation-related pathways, demonstrating significantly superior inhibitory effects compared to other mitochondria-targeting drugs [63]. Additionally, TEAD4, as the core DNA-binding protein in the YAP transcriptional complex, regulates the expression of mitochondrial-encoded electron transport chain genes in a YAP-dependent manner, directly affecting the function of the mitochondrial respiratory chain [64]. In hepatocellular carcinoma cells, the expression level of YAP was dose-dependently reduced by evodiamine, which induced mitochondrial dysfunction-mediated apoptosis and significantly inhibited the growth of liver cancer cells. This mechanism was closely associated with the disruption of mitochondrial respiratory chain integrity [65]. In colorectal cancer cells, YAP maintains the stability of mitochondrial respiratory chain function by suppressing the JNK-Drp1-mediated mitochondrial fission pathway, reducing the release of mitochondrial apoptotic factors and promoting tumor cell survival and migration [60]. These studies demonstrate that YAP/TAZ participate in the reprogramming of tumor cell energy metabolism by influencing respiratory chain gene expression and mitochondrial structural stability, thereby conferring metabolic advantages for tumor development.

**Figure 5:**
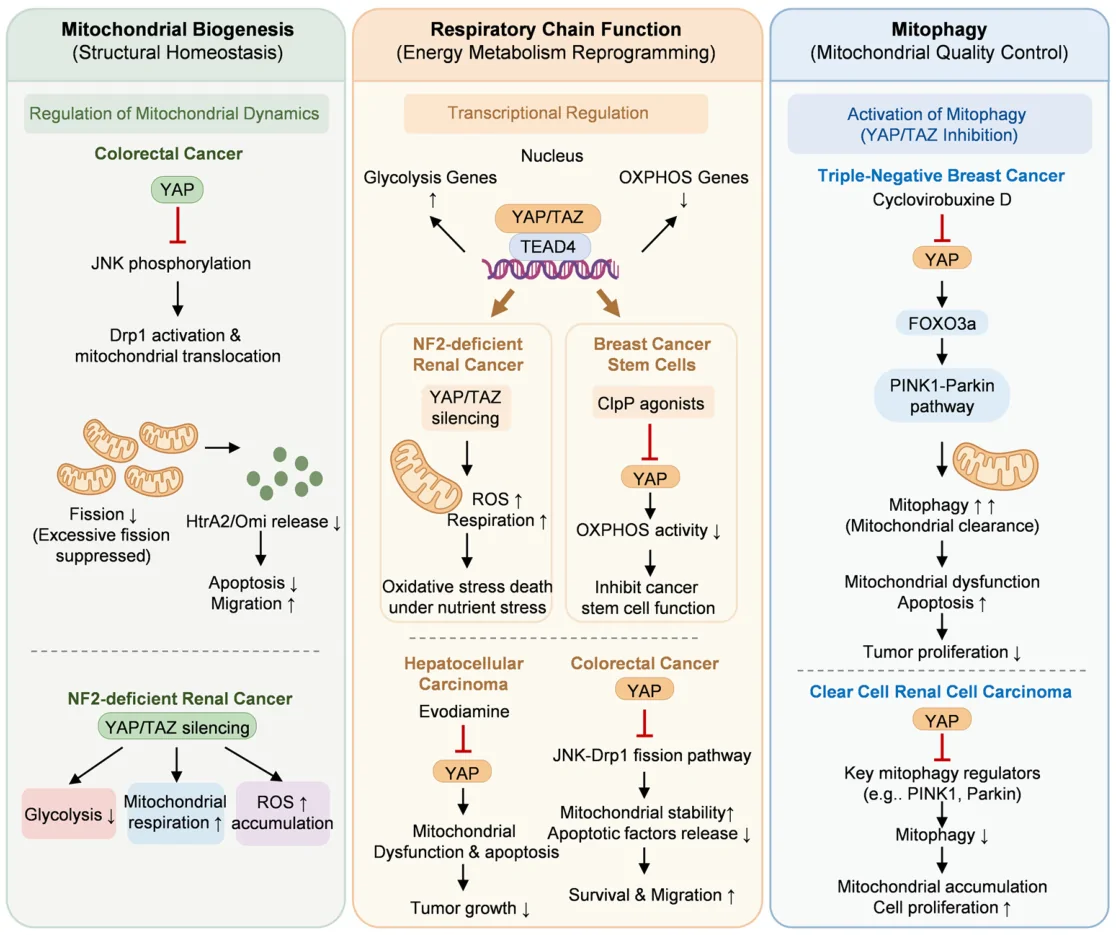
YAP/TAZ-mediated regulation of mitochondrial homeostasis and metabolic reprogramming in cancer. YAP/TAZ regulate mitochondrial function through three major mechanisms, including mitochondrial dynamics, respiratory chain activity, and mitophagy. YAP activation suppresses JNK–Drp1-mediated mitochondrial fission, reduces mitochondrial apoptotic factor release, and promotes tumor cell survival and migration. In NF2-deficient renal cancer, YAP/TAZ inhibition disrupts metabolic homeostasis by reducing glycolysis, enhancing mitochondrial respiration, and increasing ROS accumulation, leading to oxidative stress-induced cell death. Through TEAD-dependent transcriptional regulation, YAP/TAZ modulate glycolytic and OXPHOS-related gene expression to reshape mitochondrial energy metabolism. In addition, YAP/TAZ inhibit mitophagy to maintain mitochondrial stability, whereas YAP inhibition activates mitophagy-associated pathways and promotes mitochondrial dysfunction. Collectively, YAP/TAZ coordinate mitochondrial homeostasis and metabolic adaptation, thereby facilitating tumor progression. The effects of YAP/TAZ on mitochondrial dynamics are context-dependent and vary across tumor types.

Mitophagy, as a crucial mechanism for selectively clearing damaged mitochondria to maintain cellular metabolic homeostasis, is negatively regulated by YAP/TAZ. In triple-negative breast cancer, cyclovirobuxine D directly binds to YAP and inhibits its nuclear translocation, while activating the FOXO3a/PINK1-Parkin pathway to induce excessive mitophagy, leading to mitochondrial dysfunction and ultimately suppressing tumor cell proliferation and inducing apoptosis [66]. Furthermore, high TAZ expression suppresses mitophagy activity by inhibiting the expression of key mitophagy regulators, thereby promoting the proliferation of clear cell renal cell carcinoma [67]. These studies confirm that YAP/TAZ participate in the reprogramming of tumor cell energy metabolism by inhibiting mitophagy activity. Their aberrant expression disrupts mitochondrial homeostasis and metabolic balance, enabling tumor cells to adapt to microenvironmental conditions such as hypoxia and nutrient deprivation. In summary, YAP/TAZ form a complex regulatory network with tumor cell mitochondria by multi-dimensionally regulating mitochondrial biogenesis, respiratory chain function, and mitophagy, thereby influencing energy metabolism patterns and biological behaviors (Figure 5).

## 7. Metabolic Factors Regulate YAP/TAZ Activity

YAP/TAZ extensively regulate glucose, lipid, amino acid, and nucleotide metabolism through transcriptional reprogramming, providing tumor cells with the energy and biosynthetic precursors required for proliferation. Conversely, fluctuations in metabolite concentrations within tumor cells can also modulate YAP/TAZ activity, forming a bidirectional feedback loop. Glucose, lactate, fatty acids and their derivatives, amino acids, as well as metabolic factors derived from cancer-associated fibroblasts (CAFs) in the tumor microenvironment, can all influence the nuclear localization, transcriptional activity, and protein stability of YAP/TAZ through direct or indirect mechanisms, thereby contributing to tumor progression [11].

Glucose concentration serves as a critical metabolic signal regulating YAP/TAZ activity, with high-glucose environments enhancing YAP/TAZ activity through multiple signaling axes. When tumor cells actively uptake glucose and undergo glycolysis, YAP/TAZ remain fully activated, whereas their transcriptional activity significantly decreases when glucose metabolism is impaired or glycolysis is reduced. This demonstrates that glucose is an essential requirement for YAP/TAZ to exert their pro-tumorigenic functions. The underlying mechanism involves the glycolytic key enzyme phosphofructokinase-1 (PFK1), which can directly bind to TEAD and promote functional cooperation between YAP/TAZ and TEAD [68]. In colorectal cancer patients, those with concurrent type 2 diabetes exhibit significant upregulation of the TEAD/YAP/TAZ pathway in colonic mucosal cells, suggesting that the hyperglycemic microenvironment associated with diabetes may initiate local carcinogenesis through activation of this pathway [69]. In pancreatic cancer, hyperglycemia induces metabolic reprogramming toward a glycolytic phenotype, promotes epithelial–mesenchymal transition via the YAP/TAZ-Hedgehog signaling axis, and upregulates ABCB1 to enhance chemoresistance [19]. Moreover, glucose uptake itself participates in YAP/TAZ regulation. As discussed in Section 2, the GLUT3-YAP positive feedback loop exemplifies how glucose uptake itself sustains YAP/TAZ activity, creating a self-reinforcing circuit that amplifies glycolytic flux and malignant phenotypes in colorectal cancer [70]. Hypoglycemic stress suppresses YAP/TAZ through metabolic reprogramming. In pancreatic cancer cells adapted to acidic microenvironments, increased glucose uptake but weakened glycolysis shifts metabolic flux toward the pentose phosphate pathway, with elevated ATP levels inhibiting AMPK, thereby activating the YAP/MMP1 axis to promote tumor progression [71]. In addition, post-translational modifications of YAP are also involved in glucose-dependent regulation. In lung cancer cells, high glucose conditions enhance ISGylation of YAP, inhibit its ubiquitin-dependent degradation, and promote transcriptional activation of PPP key enzyme glucose-6-phosphate dehydrogenase [72].

Lipids and their metabolites also feedback-regulate YAP/TAZ activity. Unsaturated fatty acids produced during fatty acid synthesis can remodel the cellular actin cytoskeleton, inhibit LATS1/2 kinase activity, reduce YAP phosphorylation, and promote its nuclear accumulation, ultimately leading to tumor progression and resistance to lenvatinib [73,74]. The intermediate product geranylgeranyl pyrophosphate (GGPP) of the mevalonate pathway can activate YAP/TAZ by stimulating Rho GTPases, forming a pathway-specific positive feedback loop that further enhances cholesterol synthesis in hepatocellular carcinoma (Figure 2) [34,75]. The cholesterol metabolism intermediate 2,3-oxidosqualene (catalytically produced by squalene epoxidase SQLE) can bind to integrins, promoting YAP nuclear accumulation and YAP/TEAD-dependent gene expression, thereby accelerating tumor growth and metastasis [76]. Additionally, diacylglycerol lipase α (DAGLA) inhibits the phosphorylation of LATS1 and YAP through the production of 2-arachidonoylglycerol, facilitating YAP nuclear translocation and activating the PI3K/AKT pathway [73]. In the ovarian cancer microenvironment, abundant polyunsaturated fatty acids reduce YAP1 nuclear localization by inhibiting RhoA-GTPase activity, promoting the polarization of tumor-associated macrophages toward the M2 phenotype and forming an immunosuppressive microenvironment [77]. Lactate, as the end product of glycolysis, accumulates extensively in tumor cells to create an acidic microenvironment and can regulate YAP/TAZ activity through multiple mechanisms. In hepatocellular carcinoma, LDHA promotes lactate production and mediates lactylation of lysine 102 on YAP, inhibiting its Ser127 phosphorylation and thereby further facilitating malignant progression [78]. Under chronic acidosis conditions, pancreatic cancer cells enhance the activity of the pentose phosphate pathway, and elevated ATP levels inhibit AMPK, thereby activating the YAP/MMP1 axis to promote tumor progression [71]. The lipoprotein receptor LSR can bind to YAP1 through its PPPY motif, promoting the phosphorylation of YAP1 at serine 127, and inhibiting its nuclear translocation and transcriptional activity. Conversely, the absence of LSR leads to YAP1 activation, indirectly affecting the expression of genes related to cholesterol uptake [79].

Beyond the shared regulatory mechanisms, TAZ stability is also modulated by glucose metabolism through a unique moonlighting function of glucokinase (GCK). Under hypoxic conditions, CK2-mediated phosphorylation of GCK promotes its nuclear translocation, where GCK functions as a protein kinase to directly phosphorylate TAZ at Thr346. This phosphorylation recruits PIN1 for cis-trans isomerization of TAZ, preventing β-TrCP-mediated ubiquitination and degradation, thereby sustaining TAZ protein levels and downstream TEAD-mediated transcription [80]. This non-canonical moonlighting function of a glycolytic enzyme bridging glucose metabolism to TAZ stabilization adds another layer of metabolic feedback regulation.

Amino acid metabolites are also involved in the regulation of YAP/TAZ activity (Figure 3). Glutamine deficiency can overturn the conventional view that nutrient deprivation suppresses YAP. Impaired glutamine metabolism leads to increased reactive oxygen species, which inhibits LATS kinase activity via RhoA, inducing YAP dephosphorylation and nuclear translocation, thereby activating its transcriptional activity. Activated YAP directly regulates ATF4 transcription through TEAD, subsequently inducing Sestrin2 expression. The latter promotes cell survival under glutamine-deficient conditions by inhibiting mTORC1 activity [81]. Serine stabilizes the YAP through USP7-mediated deubiquitination. Under high serine conditions, the YAP-USP7 interaction is enhanced, preventing YAP ubiquitination and degradation, thereby upregulating target genes such as CTGF and CyclinD1 to promote cancer cell proliferation [44].

D-ribose-5-phosphate (D5P) as a metabolic checkpoint for YAP. Beyond amino acid metabolites, the pentose phosphate pathway (PPP) intermediate D-ribose-5-phosphate (D5P) functions as a key metabolic checkpoint that directly suppresses YAP activity [50]. Mechanistically, D5P directly binds to the MYH9 protein, sterically hindering its association with LATS1. This sequestration prevents MYH9-mediated LATS1 oligomerization and subsequent ubiquitin–proteasome degradation, thereby maintaining LATS1 kinase activity and promoting YAP phosphorylation at Ser127 to inhibit its transcriptional activity. Under glucose deprivation, cellular D5P levels drop markedly, releasing MYH9 from this inhibition. Liberated MYH9 then interacts with LATS1, promoting its aggregation and degradation, which relieves YAP phosphorylation and triggers YAP nuclear translocation and transcriptional activation. Notably, activated YAP in turn upregulates PNP to enhance purine degradation and replenish D5P, forming a closed homeostatic feedback loop that enables tumor cells to adapt to nutrient stress by fine-tuning both nucleotide metabolism and YAP activity [50].

The stromal components in the tumor microenvironment, particularly CAFs, regulate YAP/TAZ activity by secreting various metabolites and cytokines. The extracellular matrix components of CAFs and factors secreted by tumor cells can activate the YAP/TAZ pathway through mechanical signals. For example, pancreatic cancer cells secrete tissue transglutaminase (TG2), which remodels the matrix by cross-linking collagen, enhances matrix stiffness, and activates fibroblasts. These activated fibroblasts then secrete ECM components that promote tumor cell proliferation by activating YAP/TAZ through mechanical signals [82]. Under high-glucose conditions, CAFs increase the production of collagen-related proteins (such as fibronectin and COL1A1) in a YAP/TAZ-dependent manner, constructing a pro-tumorigenic stromal microenvironment [19]. Cytokines secreted by CAFs can also directly regulate YAP/TAZ activity in tumor cells. The downstream effector molecule Dickkopf-3 of HSF1 in CAFs activates the canonical Wnt signaling pathway by interfering with Kremen protein function, thereby promoting YAP/TAZ activation and inducing the pro-tumorigenic phenotype of CAFs [83]. Hepatocyte growth factor (HGF) secreted by pancreatic CAFs activates the c-MET receptor, promoting YAP nuclear translocation and HIF-1α stabilization, which subsequently upregulates HK2 to enhance glycolysis and increases stemness markers NANOG and OCT-4 to reinforce cancer stem cell properties [84]. In addition, CAFs enhance aspartate production through glycolysis, which is taken up and utilized by tumor cells, while tumor cell-secreted glutamate regulates the redox balance of CAFs and promotes their matrix remodeling function. This metabolic crosstalk relies on YAP/TAZ-mediated signaling activation [85]. YAP/TAZ themselves play a crucial role in the activation and functional maintenance of CAFs. Abnormal activation of YAP/TAZ in CAFs promotes their proliferation and secretion of pro-tumorigenic factors, while loss of YAP/TAZ signaling significantly suppresses the activated phenotype of CAFs [86]. Increased matrix stiffness enhances aerobic glycolysis in CAFs through the MAPK-YAP signaling axis, and its metabolites (e.g., lactate) act on tumor cells to activate the YAP/TAZ pathway, forming a regulatory loop of “matrix stiffness-CAF metabolism-YAP/TAZ” that accelerates tumor progression [86,87].

Beyond the metabolic cues arising from nutrients and stromal cells, changes in the physicochemical microenvironment, particularly oxygen availability, also serve as critical metabolic signals that feedback-regulate YAP/TAZ activity. Hypoxia modulates YAP/TAZ activity through both HIF-dependent and HIF-independent mechanisms, adding another layer of metabolic feedback regulation. Under hypoxic conditions, HIF-1α can directly bind to the promoter region of YAP, transcriptionally upregulating YAP expression and promoting its oncogenic functions [88]. Concurrently, hypoxia-induced reactive oxygen species (ROS) accumulation inactivates LATS1/2 kinases via oxidation or RhoA-mediated pathways, leading to YAP/TAZ dephosphorylation and nuclear accumulation [89]. In the context of lipid metabolism, hypoxia-induced HIF-2α selectively promotes the transcription of genes involved in lipid droplet formation and fatty acid uptake, which synergize with YAP/TAZ-driven lipogenesis to sustain membrane biosynthesis under oxygen-deprived conditions [90]. Moreover, HIF-1α and YAP/TAZ can co-occupy chromatin at metabolic gene loci (e.g., GLUT1, HK2, LDHA) in a TEAD-dependent or -independent manner, orchestrating a coordinated transcriptional program that maximizes glycolytic flux while suppressing oxidative phosphorylation. This reciprocal regulation not only enables tumor cells to adapt to hypoxic stress but also creates metabolic dependencies (e.g., on glutamine anaplerosis and de novo pyrimidine synthesis) that can be therapeutically exploited. Therefore, targeting the HIF-YAP/TAZ reciprocal axis may offer a promising strategy to disrupt the adaptive metabolic reprogramming of tumors in their hypoxic niche.

**Figure 6:**
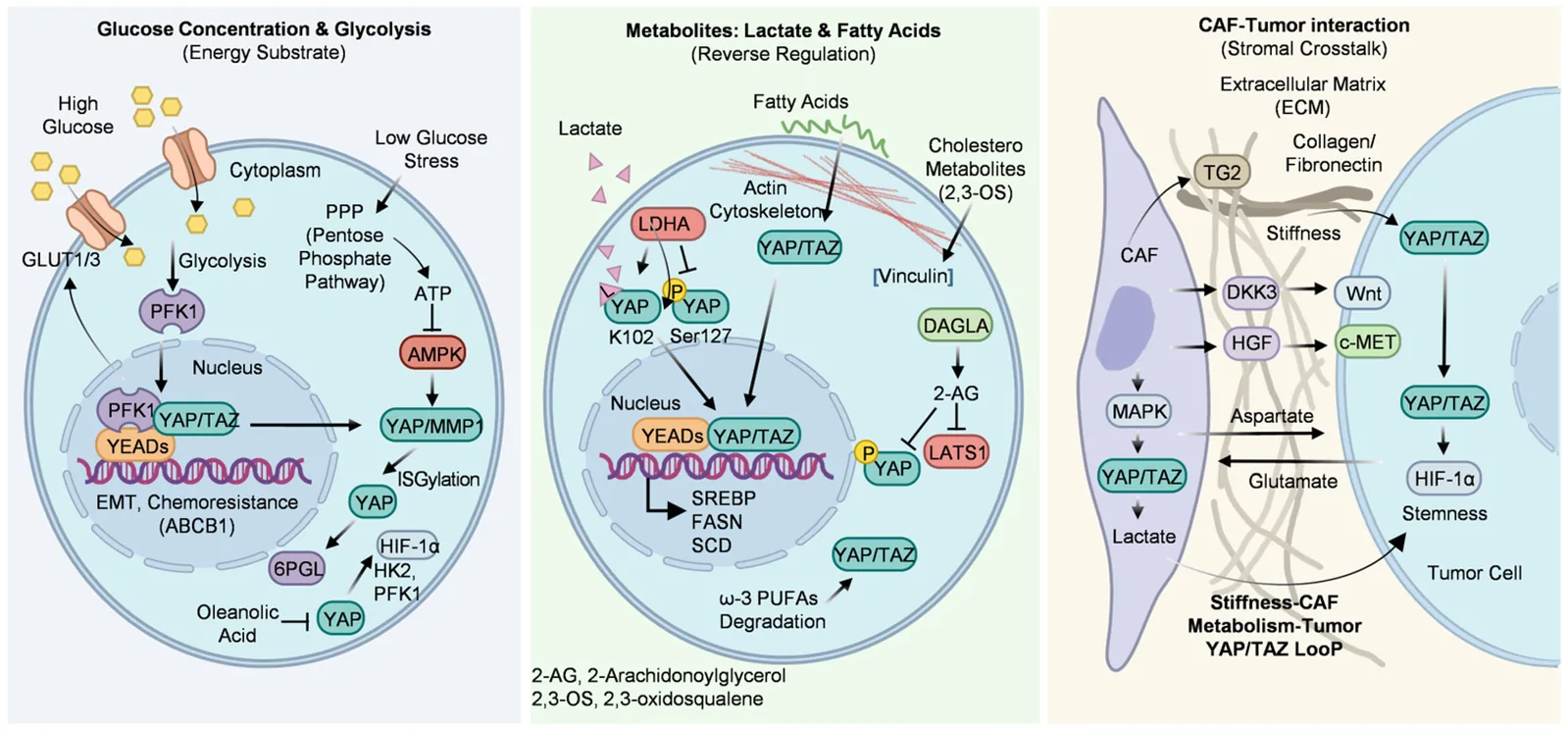
Feedback regulation of YAP/TAZ by metabolic factors. High glucose enhances YAP/TAZ activity via glycolytic enzymes (PFK1) or GLUT1/3 positive feedback loops, driving the Warburg effect. Hypoglycemic stress inhibits YAP/TAZ via metabolic flux shifts. Lactate mediates YAP lactylation, blocking Ser127 phosphorylation to boost activity. Fatty acids remodel the actin cytoskeleton to regulate YAP nuclear translocation, affecting fission/fusion control. CAF-derived factors activate YAP/TAZ via ECM remodeling or cytokine secretion, forming a positive loop (YAP/TAZ maintain CAF activation) that enhances mitophagy regulation to support progression.

In summary, fluctuations in glucose concentration, abnormal lipid metabolism, changes in amino acid levels, CAF-derived metabolic factors, as well as hypoxia/HIF signaling, can all feedback-regulate YAP/TAZ activity through direct or indirect mechanisms, forming a complex “metabolism-signaling” regulatory network involved in malignant phenotypes such as tumor proliferation, invasion, drug resistance, and immune suppression (Figure 6).

## 8. Targeting the YAP/TAZ-Metabolism Axis for Combination Cancer Therapy

### 8.1. Direct Targeting of the YAP/TAZ-TEAD Transcriptional Complex

The most direct strategy to interrupt the oncogenic function of YAP/TAZ is to target their core transcriptional complex—YAP/TAZ-TEAD. In recent years, significant progress has been made in the development of drugs targeting this complex (Table 1). According to the mechanism of action, these compounds can be classified into the following categories: directly inhibiting the protein–protein interaction of YAP-TEAD (such as verteporfin) [91]; inhibiting the transcriptional activity of TEAD (such as CA3) [92]; targeting the central hydrophobic pocket of TEAD (such as flufenamic acid and its derivatives) [93]; and covalent or non-covalent inhibition of TEAD’s own palmitoylation (such as TED-347, VT series compounds, and K-975) [94,95]. Moreover, new strategies are constantly emerging, including peptide inhibitors (such as TBDi, Peptide 17), cyclic peptides (such as Cyclic YAP-like peptides) [96,97], and molecules using PROTAC technology to degrade YAP or TEAD proteins (such as KG-FP-003, YZ-6) [98,99]. Among them, small molecule inhibitors targeting the TEAD palmitoylation pocket have made particularly rapid progress. They occupy the palmitic acid binding pocket, allosterically inhibit the function of TEAD, and thereby block the transcription of downstream oncogenic genes [100,101].

Several compounds have advanced to clinical trials (Table 2). Among them, VT3989 and IAG933 are representative drugs with relatively advanced progress. VT3989 is a first-in-class oral TEAD auto-palmitoylation inhibitor [102]. A phase I/II study involving 172 patients with refractory solid tumors showed that at the optimized clinical dose, VT3989 achieved a 26% objective response rate in patients with mesothelioma, with significant disease control and controllable safety [103,104]. IAG933 is a non-covalent oral inhibitor that directly disrupts the YAP-TEAD interaction. Its first-in-human study in 136 advanced patients (over 80% were mesothelioma) showed that the objective response rate in patients with malignant pleural mesothelioma was 16.6% at a specific dose level, and most patients achieved disease stability [105,106]. The early clinical data of these two drugs preliminarily verified the therapeutic potential of targeting TEAD in tumors with Hippo pathway dysregulation (such as mesothelioma).

As clinical exploration progresses, a new generation of TEAD inhibitors is gradually entering clinical trials. For instance, ISM6331 is a novel non-covalent TEAD inhibitor designed using artificial intelligence and has obtained orphan drug status from the FDA. It is currently undergoing Phase I clinical studies in both China and the United States for advanced malignant mesothelioma and other solid tumors [107]. Another investigational new drug, TYK-01054, is a new oral small molecule YAP/TEAD inhibitor. Its Phase I/II clinical study targeting locally advanced or metastatic solid tumors has also been initiated (NCT07282873). The continuous emergence of these new drugs not only enriches the drug arsenal targeting the Hippo pathway but also provides new possibilities for overcoming tumor resistance.

In summary, the development of small molecule drugs targeting the YAP/TAZ-TEAD axis is rapidly moving from preclinical research to clinical validation. Despite challenges (such as the termination of the IK-930 project), the positive data of VT3989, IAG933, etc., have greatly boosted confidence in this field. With more new molecules such as ISM6331 and TYK-01054 entering clinical trials, combined with a deeper understanding of biomarkers (such as NF2 mutation status), it will help achieve more precise patient stratification and treatment, and promote the application of targeted Hippo pathway therapy in precision oncology [92]. Drug information listed in Table 1 and Table 2 was compiled from Web of Science (https://www.webofscience.com) and ClinicalTrials.gov (https://clinicaltrials.gov), with data accessed between January and June 2026. Clinical trial status was verified as of August 2026.

### 8.2. Targeting Upstream Metabolic Signals to Enhance YAP/TAZ Inhibition

Given that metabolites regulate YAP/TAZ activity, pharmacological intervention to alter the metabolic microenvironment represents an indirect yet potent strategy to suppress YAP/TAZ function. Furthermore, combining such metabolic modulators with direct YAP/TAZ inhibitors can cut off upstream activating signals while simultaneously blocking the remaining transcriptional activity, offering a synergistic therapeutic approach.

High blood glucose conditions have been proven to activate YAP through various mechanisms [69]. The AMPK activated by energy stress is a negative regulator of YAP, and metformin, as a classic AMPK activator, can induce YAP phosphorylation and inactivate it [108]. Combining metformin with YAP inhibitors can not only inhibit YAP through metabolic stress but also directly block the remaining YAP activity. This has been shown to have a synergistic effect in models such as endometrial cancer [109]. Lactic acid-induced activation of YAP provides a novel approach for targeted therapy [78]. Lactic acid is not only a product of glycolysis but also a signaling molecule that activates YAP. Therefore, the combined use of lactate dehydrogenase A (LDHA) inhibitors or lactate transporters (monocarboxylate transporter 4) inhibitors can block the production or uptake of lactic acid, thereby inhibiting the activation of YAP [78]. This strategy can be used in combination with YAP inhibitors and is expected to overcome the resistance of single therapy. In addition, the mevalonate pathway serves as a crucial hub connecting lipid metabolism and YAP activation. The metabolite GGPP produced by this pathway promotes YAP nuclear translocation by activating Rho GTPases [34]. Therefore, statins, as HMGCR inhibitors, not only inhibit tumor growth by reducing cholesterol synthesis, but also suppress YAP activity by reducing GGPP production. Combining statins with YAP/TEAD inhibitors can block the YAP signal at both the metabolic and transcriptional levels.

These strategies highlight the potential of targeting metabolic inputs that sustain YAP/TAZ activity. Combining upstream modulators with direct YAP/TAZ-TEAD complex inhibitors may achieve more profound and durable therapeutic responses.

### 8.3. Combination Strategies Targeting YAP/TAZ-Regulated Metabolic Effectors

Given the extensive crosstalk between YAP/TAZ and cellular metabolism, and the fact that YAP/TAZ regulate the expression of key metabolic enzymes to promote cancer development, combining YAP/TAZ inhibition with strategies targeting these downstream metabolic vulnerabilities has emerged as a promising direction. Recent studies have shown that simultaneously disrupting YAP/TAZ and its key metabolic effectors can produce a synergistic anti-tumor effect, opening up a promising avenue for future cancer treatment.

In terms of glucose metabolism, YAP upregulates the expression of multiple key glycolytic enzymes through transcriptional regulation or protein stability, such as HK2, PKM2 and LDHA. Therefore, the combined use of YAP inhibitors and glycolysis inhibitors can produce a synergistic effect. For example, in gastric cancer, the SGLT2 inhibitor dapagliflozin has been proven to promote the degradation of YAP1 by regulating OTUD5, thereby inhibiting glycolysis [110]. This suggests that the combined application of dapagliflozin with YAP inhibitors (such as verteporfin) may have synergistic therapeutic potential in YAP-driven tumors. Additionally, targeting HK2 downstream of YAP is also a reasonable strategy. Studies have shown that a HK2-specific inhibitor (such as 2-deoxy-D-glucose, which competes with glucose for hexokinase binding) can enhance the sensitivity of tumor cells to chemotherapy [111]. In terms of lipid metabolism, tumors with high YAP activity exhibit a high dependence on fatty acid and cholesterol synthesis. YAP cooperates with SREBPs to upregulate the expression of key lipid synthesis enzymes such as FASN, ACC, and SCD [29,30]. This provides a theoretical basis for combined treatment. The commonly used statin drugs (such as atorvastatin), as HMGCR inhibitors, show stronger anti-tumor effects in colorectal cancer with high YAP expression, suggesting that YAP activity can be used as a biomarker for the treatment with statins [34]. In terms of amino acid and nucleotide metabolism, YAP/TAZ also regulate multiple key nodes. YAP promotes glutamine uptake by upregulating transporters such as SLC1A5 and induces the expression of metabolic enzymes such as glutaminase 1 and GLUL [37,38]. It is noteworthy that in nucleotide synthetic metabolism, YAP can directly transcriptionally upregulate the key enzymes of pyrimidine synthesis, CAD and DHODH, especially in NF2-deficient mesothelioma, which makes tumor cells highly sensitive to pyrimidine synthesis inhibition [54]. Therefore, for tumors with NF2 deficiency (abnormal activation of YAP), the combined use of YAP inhibitors (or TEAD inhibitors) and DHODH inhibitors (such as leflunomide or more efficient Brequinar) may achieve efficient and low-toxic anti-tumor effects by doubly blocking the transcriptional function of YAP and its downstream pyrimidine synthesis.

**Table 1:** Summary of representative compounds targeting YAP/TAZ-TEAD.

Compound	Mechanism	Inhibitor Type	Reference
Verteporfin	Inhibit the interaction between YAP and TEAD	Small molecule inhibitors	[91]
CA3 (CIL56)	Effectively inhibit the transcriptional activity of YAP1/TEAD	Small molecule inhibitors	[112]
Flufenamic acid	Inhibit transcriptional activity by integrating into the central hydrophobic pocket of TEAD	Small molecule inhibitors	[93]
TED-347	Inhibit the YAP-TEAD complex by combining with the palmitic acid pocket of TEAD	Small molecule inhibitors	[113]
VT103	Inhibition of the auto-palmitoylation of TEAD proteins	Small molecule inhibitors	[94]
VT104	Inhibition of the auto-palmitoylation of TEAD proteins	Small molecule inhibitors	[94]
VT107	Inhibition of the auto-palmitoylation of TEAD proteins	Small molecule inhibitors	[94]
MSC-4106	Inhibition of the auto-palmitoylation of TEAD proteins	Small molecule inhibitors	[94]
K-975	Covalently binding TEAD to inhibit the YAP/TAZ-TEAD interaction	Small molecule inhibitors	[95]
GNE-7883	Pan TEAD inhibitor, blocking the binding of YAP/TAZ to TEAD	Small molecule inhibitors	[114]
HC-258	Covalently bound to the palmitic acid pocket of TEAD	Small molecule inhibitors	[115]
MGH-CP1	Selective TEAD palmitoylation inhibitor, inhibition of TEAD2/4	Small molecule inhibitors	[116]
MYF-01-37	Covalent inhibitor of Cys380 targeting TEAD2	Small molecule inhibitors	[117]
Cerivastatin	Promote YAP phosphorylation and modulate YAP/TAZ activity via the mevalonate pathway, thereby suppressing cancer cell self-renewal and tumor growth	Small molecule inhibitors	[5,118]
Hapalindole Q	Degradation of YAP1 by molecular chaperone-mediated autophagy	Small molecule inhibitors	[119]
Resveratrol	Inhibit YAP expression and sensitize cancer cells to chemotherapy, and activation of AMPK suppresses YAP activity by reducing its nuclear translocation	Small molecule inhibitors	[120,121]
CV-4-26	Covalent inhibitors of TEAD	Small molecule inhibitors	[122]
Alexidine	Directly targeting TAZ protein disrupts TAZ-TEAD interaction	Small molecule inhibitors	[123]
MRK-A	Targeting the palmitoylated pocket of TEAD, allosteric inhibition of YAP1/TAZ-TEAD complex. Combining a MET inhibitor with the YAP1/TEAD antagonist MRK-A overcomes HGF-induced therapeutic resistance	Small molecule inhibitors	[124]
Derivatives of Niflumic Acid (compound **5a**, **5b** and **5k**)	Combined with TEAD-YAP binding domain, YAP/TAZ transcriptional activity was inhibited	Small molecule inhibitors	[125]
SWTX-143	Inhibition of TEAD autopalmitoylation blocked the transcriptional output of the Hippo pathway	Small molecule inhibitors	[126]
MSC-4070	Selective inhibition of TEAD1, 2, 4, with central nervous system penetration ability	Small molecule inhibitors	[127]
Ethacrynic Acid	Occupy the TEAD palmitoylation pocket, and inhibition of YAP/TAZ-TEAD interaction	Small molecule inhibitors	[128]
AR-42	Highly effective TAZ inhibitors	Small molecule inhibitors	[129]
LM-41	Targeting TEAD	Small molecule inhibitors	[130]
AF-2112	Targeting TEAD	Small molecule inhibitors	[130]
mCMY020	Inhibition of YAP-TEAD transcription complex formation	Small molecule inhibitors	[131]
TBDi	Specific destruction of YAP-TEAD interaction	Polypeptide	[96]
Peptide 17	Inhibition of YAP-TEAD protein interaction	Polypeptide	[132]
Super-TDU	Competitively inhibit YAP and destroy YAP-TEAD interaction	Polypeptide	[133]
Cyclic YAP-like peptides	Competitively inhibit YAP and destroy YAP-TEAD interaction	Polypeptide	[97]
KG-FP-003	Efficient and selective TEAD protein degradation agent	PROTAC	[98]
E8-2RNF4	Targeted degradation of endogenous YAP	PROTAC	[134]
YZ-6	YAP is degraded by VHL E3 ligase	PROTAC	[99]

**Table 2:** YAP/TAZ-TEAD–targeting drugs in cancer clinical trials.

Phase I	Phase II	Drug	Indicated Indications	Clinical Trial	Sponsor
26 October 2023	26 October 2023	ODM-212	Neoplasms, benign, malignant and unspecified	NCT06725758, Phase I/II	Orion Corporation (Orionintie 1A, 02200 Espoo, Finland)
7 January 2022 Terminated	/	IK-930	Advanced solid tumors	NCT05228015, Phase I	Ikena Oncology (Boston, MA, USA)
24 June 2024	/	ISM6331	Advanced or metastatic malignant mesothelioma	NCT06566079, Phase I	InSilico Medicine Hong Kong Limited (Hong Kong, China)
27 April 2024	1 January 2026	TYK-01054	Locally advanced or metastatic advanced solid tumors	NCT07282873, Phase I/II	TYK Medicines, Inc. (Huzhou, China)
24 March 2021	8 October 2025	VT3989	Mesothelioma	NCT04665206, Phase I/II	Vivace Therapeutics (San Mateo, CA, USA)
5 January 2021	/	ION537	Advanced head and neck squamous cell carcinoma	NCT04659096, Phase I	Nationales Centrum für Ionis Pharmaceuticals Inc. (Carlsbad, CA, USA)
30 July 2024	/	SW-682	Advanced solid tumors	NCT06251310, Phase I	SpringWorks Therapeutics, Inc. (Stamford, CT, USA)
12 October 2021	/	IAG-933	Mesothelial tissue tumor	NCT04857372, Phase I	Novartis AG (Basel, Switzerland)
28 June 2024	/	BGC515	Advanced solid tumors	NCT06452160, Phase I	BridGene Biosciences (San Jose, CA, USA)
24 April 2023	/	BPI-460372	Advanced solid tumors	NCT05789602, Phase I	Betta Pharmaceuticals Co., Ltd. (Hangzhou, China)
2026 Preclinical (IND filed)	/	SIGX2649	Advanced solid tumors	Completed preclinical research	Signet Therapeutics (Shenzhen) Co., Ltd. (Shenzhen, China)
10 June 2024	/	ETN-006	Mesothelioma of pleura	Orphan Drug Designation	ETERN Therapeutics (Shanghai, China)

## 9. Conclusions and Perspectives

In summary, YAP/TAZ can influence various metabolic processes, including glucose, lipid, amino acid, and nucleotide metabolism, as well as mitochondria structure and function, thereby supplying the energy and synthetic materials required for the sustained proliferation of cancer cells. Simultaneously, various intracellular metabolites and metabolic signals within the tumor microenvironment can, in turn, affect the activity and localization of YAP/TAZ, forming a mutually regulatory relationship that collectively drives tumor initiation, progression, metastasis, and the development of drug resistance. Based on these interactions, targeting the YAP/TAZ-metabolic pathway has emerged as a viable anticancer strategy. Current research directions for therapeutic approaches include directly inhibiting the binding of YAP/TAZ to TEAD, blocking upstream metabolic signals that activate YAP/TAZ, and combining YAP/TAZ inhibitors with metabolic pathway inhibitors. Notably, some TEAD-targeting compounds have entered clinical trials, demonstrating efficacy in tumors dependent on Hippo pathway dysregulation, such as mesothelioma, thereby providing a basis for subsequent clinical applications. The combination of YAP/TAZ inhibitors with metabolic pathway inhibitors has also shown promising results in multiple preclinical studies. It is particularly noteworthy that, although YAP and TAZ are frequently discussed collectively, emerging evidence has revealed TAZ-specific regulatory mechanisms in glucose and lipid metabolism that operate independently of YAP. Nevertheless, mechanistic studies on YAP-independent functions of TAZ in most metabolic pathways remain extremely limited. The metabolic regulatory patterns of YAP/TAZ exhibit significant differences across various tumors and tissues, and identifying suitable biomarkers to screen eligible patients for treatment remains an unresolved challenge. Tumor cells inherently possess metabolic flexibility, readily developing drug resistance by altering metabolic pathways, thus necessitating further exploration of rational combination therapies. Additionally, the relationships among YAP/TAZ, metabolic reprogramming, the tumor microenvironment, and immune responses require more systematic research to elucidate. Future studies should systematically distinguish the differential roles of YAP and TAZ in tumor metabolic networks, which will not only deepen our understanding of the crosstalk between the Hippo pathway and cancer metabolism but may also provide a theoretical foundation for developing precision therapeutic strategies that selectively target YAP or TAZ.

Future studies should focus on improving the clinical applicability of YAP/TAZ-targeted strategies by addressing several unresolved issues. A major challenge is the identification of reliable biomarkers that can define tumors with strong YAP/TAZ dependence or specific metabolic vulnerabilities, thereby enabling more precise patient stratification. In addition, considering the extensive metabolic adaptation induced by YAP/TAZ activation, combination therapies integrating YAP/TAZ inhibitors with agents targeting key metabolic pathways may represent a promising approach to overcome therapeutic resistance. However, the optimal combination strategies and their context-dependent effects require further validation in clinically relevant models. Emerging technologies, including single-cell sequencing, spatial transcriptomics, and metabolomic profiling, may provide additional insights into the heterogeneity of YAP/TAZ-driven metabolic programs and their interactions with the tumor microenvironment. Ultimately, a deeper understanding of these mechanisms will be critical for translating YAP/TAZ–metabolism-based interventions into effective precision cancer therapies.

Overall, YAP/TAZ play a pivotal role in tumor metabolic reprogramming, and their mutual regulation with metabolic pathways constitutes a crucial aspect in understanding the mechanisms of cancer development. Continued exploration of this network will not only contribute to refining the fundamental theories of cancer, but also provide novel insights for drug development and personalized therapy. As research advances, therapeutic strategies targeting the interplay between YAP/TAZ and metabolism are expected to emerge as effective clinical options for treating advanced and drug-resistant tumors.

## Data Availability

No new data were created or analyzed in this study. Data sharing is not applicable to this article.

## Abbreviations

The following abbreviations are used in this manuscript:

ACC	Acetyl-CoA carboxylase
AMPK	AMP-activated protein kinase
CAF	Cancer-associated fibroblast
CAD	Carbamoyl–phosphate synthetase 2, aspartate transcarbamylase, and dihydroorotase
CPT1A	Carnitine palmitoyltransferase 1A
DHODH	Dihydroorotate dehydrogenase
Drp1	Dynamin-related protein 1
FAO	Fatty acid oxidation
FASN	Fatty acid synthase
GGPP	Geranylgeranyl pyrophosphate
GLUL	Glutamine synthetase
GLUT	Glucose transporter
HIF-1α	Hypoxia-inducible factor 1α
HK2	Hexokinase 2
HMGCR	3-Hydroxy-3-methylglutaryl-CoA reductase
LDHA	Lactate dehydrogenase A
mTORC1	Mechanistic target of rapamycin complex 1
ODC1	Ornithine decarboxylase 1
PHGDH	Phosphoglycerate dehydrogenase
PKM2	Pyruvate kinase M2
PPP	Pentose phosphate pathway
ROS	Reactive oxygen species
SLC	Solute carrier family
SREBP	Sterol regulatory element-binding protein
TEAD	TEA domain transcription factor
